# Impact of Fixation Length for Single-Level Spinal Metastasis Surgery in the Elderly: A Multicenter Study

**DOI:** 10.7759/cureus.80886

**Published:** 2025-03-20

**Authors:** Hiroki Ito, Yasuhiko Takegami, Hiroaki Nakashima, Kenichi Mishima, Kenichi Yamauchi, Shiro Imagama

**Affiliations:** 1 Department of Orthopaedic Surgery, Nagoya University Graduate School of Medicine, Nagoya, JPN; 2 Department of Orthopaedic Surgery, Toyohashi Municipal Hospital, Toyohashi, JPN

**Keywords:** complications, minimally invasive surgery, spinal fixation surgery, spinal metastasis, survival

## Abstract

Purpose

Spinal metastases are common and affect most patients with metastatic cancer. Surgery is crucial for managing symptoms such as pain and paralysis but carries a high risk of complications, especially in the elderly. This study aimed to investigate the impact of short versus long fixation on outcomes mainly focused on surgery-related complications in elderly patients ≥60 years old undergoing surgery for single-level spinal metastasis.

Methods

This study is a retrospective multicenter cohort study. We retrospectively analyzed data from 153 patients who underwent spinal metastasis surgery between 2014 and 2023, focusing on 44 patients aged ≥60 years with single-level lesions. Out of 151 patients, those under 60 years old, those who underwent only decompression surgery, and those with missing data were excluded, resulting in a final inclusion of 44 patients. Patients were divided into short fusion (four or fewer vertebrae) and long fusion (five or more vertebrae) groups. This study aims to compare the impact of short (four or fewer vertebrae) versus long (five or more vertebrae) spinal fixation on surgical and patient-centered outcomes in elderly patients undergoing surgery for single-level spinal metastasis. Patients’ demographics, surgical details, and complications were compared between the groups.

Results

The study included 44 patients: long fusion group (n = 24) and short fusion group (n = 20). No significant between-group differences were found in demographics or surgery-related factors. Minimally invasive surgery was performed in 29.2% of long fusion group patients and 10% of short fusion group patients, but this difference was not statistically significant. Survival analysis showed no significant difference in median survival between the groups (short fusion group = 29 months vs. long fusion group = 26 months, P = 0.533). Mortality rates were also similar between groups during the follow-up period. The short fusion group had a higher rate of surgical complications (45% vs. 12.5%, P = 0.021) and more frequent postoperative complications (55% vs. 29.2%, P=0.125), although the difference was not statistically significant.

Conclusion

This study found no significant difference in survival rates between short and long fixation in elderly patients undergoing surgery for single-level spinal metastasis. In spinal metastasis surgery, extending the fixation range may be considered if necessary.

## Introduction

The spine is the most common site of bone metastasis, with studies indicating that the axial skeleton is affected in up to 50-70% of patients with metastatic cancer [1,2]. Reports reveal that approximately one-third of patients diagnosed as having cancer will develop spinal metastases during the course of their disease [3,4]. Spinal metastases are often asymptomatic, but severe pain and paralysis may occur as they progress [5].

Surgery plays a critical role, particularly in cases in which progressive neurological symptoms or spinal instability threaten the patient’s quality of life [6]. However, spine surgery for metastatic spinal tumors is known to carry a high risk of complications. A previous study reported that morbidity can be relatively high, with complication rates of 34.0% in patients who underwent surgical intervention [7]. Even in relatively young patients, there is a clear link between complications and mortality [8].

In elderly patients, the decision to pursue surgical intervention must be carefully weighed against the increased risks associated with age-related comorbidities [9]. Previous studies have reported that advanced age is a risk factor for complications in metastatic spinal tumors [10]. It has also been found that in general spinal fusion procedures, the greater the extent of fusion, the higher the rate of complications [11]. However, due to concerns such as osteoporosis, longer fixation may be chosen in elderly patients even if it is more invasive [12]. Despite the increasing incidence of spinal metastases in the aging population, few studies have specifically focused on the extent of fixation in elderly patients undergoing surgery for spinal metastases.

As reported in previous studies, we hypothesized that in elderly patients with spinal metastases, a longer fixation would provide greater stability but would also increase the incidence of complications. This study examined elderly patients ≥60 years old who had surgery for single-level spinal metastasis to compare the effects of short fixation and longer fixation methods on survival and complications in a multicenter study, which includes the seven hospitals of the Trauma Research of Nagoya (TRON) group.

## Materials and methods

Subjects

**Figure 1:**
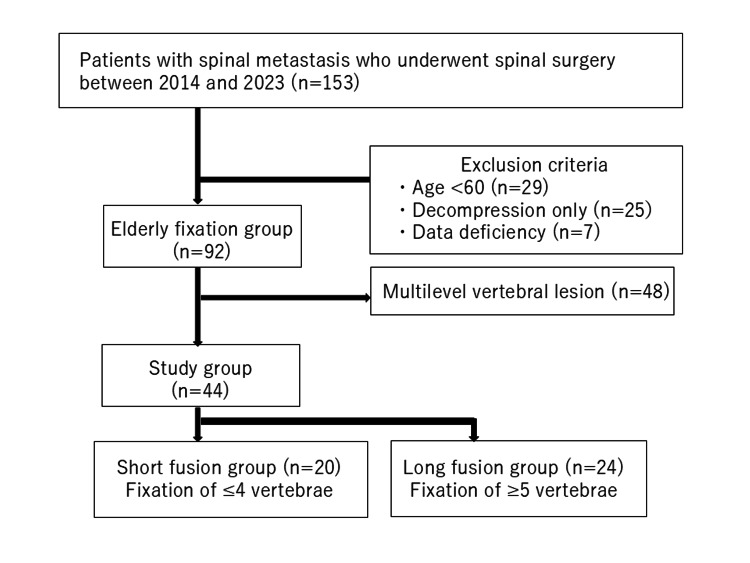
Flow diagram of the participants included in the study.

Data retrospectively collected from a database were used to identify 153 patients who underwent surgery for spinal metastasis between 2014 and 2023. Surgical indications included conditions causing neurological deficit or pain and concern for instability such as having a high spinal instability neoplastic (SIN) score [13]. The extent of spinal fixation during surgery was determined by the surgeon. This study focused on patients with a single-level vertebral lesion. Exclusion criteria were patients younger than 60 years old at the time of surgery, those who did not undergo spinal fusion surgery, and those with multilevel vertebral lesions. As shown in Figure 1, 153 patients were initially included. Those who met the exclusion criteria, including patients under 60 years old (n = 29), those who underwent only decompression surgery (n = 25), those with incomplete data (n = 7), and those with multiple vertebral lesions (n = 48), were excluded. Ultimately, 44 patients aged 60 years or older who underwent fixation surgery for a single vertebral lesion were included. The patients were divided into two groups: the long fusion group with fixation of five or more vertebrae and the short fusion group with fixation of four or fewer vertebrae. We examined elderly patients ≥60 years old who had surgery for single-level spinal metastasis to compare the effects of both short fixation and longer fixation on survival and complications.

Data collection

All patients included in this study had a diagnosis of metastasis by bone biopsy or intraoperative pathological examination. The demographic data, which were collected retrospectively from each patient’s medical records, included age, gender, body mass index (in kg/m2), comorbidity status, Eastern Cooperative Oncology Group performance status [14], cancer type, location of spine metastatic lesion, bone metastases outside the spine, visceral metastases (lung or liver), the time between neurologic deficits and surgery, location of the metastatic lesion, prior radiotherapy to the spinal tumor, prior systemic therapy for the cancer diagnosis (all non-surgical and non-radiotherapeutic adjuvants), neurologic status before surgery and at discharge using the Frankel grade [15], type of surgery and approach, number of spine levels operated upon, type of instrumentation, duration of surgery (in minutes), duration of hospital stay (in days), and estimated blood loss (in milliliters). We also calculated the SIN score [13], Tokuhashi score [16], new Katagiri score [17], and Charlson Comorbidity Index (CCI) score [18].

The outcomes in this study were complications, defined as any intraoperative or postoperative event that required additional medical or surgical treatment, and mortality, which was recorded both independently and as a result of complications.

Surgical procedure

The decisions regarding the surgical treatment, surgical procedure (such as posterior fixation, number of vertebrae fused, and bone grafting), and the timing of surgery were based on the surgeon’s preference and intentions of the individual or family. Surgery was performed under general anesthesia in all cases. Posterior fixation or posterior decompression and fixation were performed in this study. For the minimally invasive surgery (MIS), only a percutaneous pedicle screw (PPS) was used for fixation, and both posterior fixation only and combined decompression were performed. In this study, MIS was defined as fixation by PPS.

Statistical analysis

Continuous variables were compared using the Fisher's exact test or Mann-Whitney U test. Survival analysis was performed using the Kaplan-Meier method with the log-rank test. Statistical significance was set at P < 0.05. All statistical analyses were performed with EZR [19], which is for R statistical software (R Foundation for Statistical Computing, Vienna, Austria). More precisely, it is a modified version of R commander designed to add statistical functions frequently used in biostatistics. No statistical sample size calculations were conducted. However, with sample sizes of 20 and 24 patients per group, the post hoc powers for comparing the proportions between the two groups regarding postoperative complication rates (during hospitalization, during follow-up, and other complications), using a two-group test with a two-sided significance level of P < 0.05 for percentage change, were 53.4%, 28.0%, and 11%, respectively. Confounding factors include the presence or absence of tumor recurrence, timing of surgery, surgical technique and experience, and prognosis. Although no differences were observed between the groups regarding tumor type, timing of surgery, or prognosis, factors such as recurrence and technical aspects were not considered in this study.

## Results

Patient background

This study included 44 elderly patients who underwent surgery for single vertebra spinal metastasis. The group that received long fusion comprised 24 patients, whereas the short fusion group comprised 20 patients. As shown in Table 1, the average age was 73.75 years old (SD = 6.94, range = 62-85 years) for the long fusion group and 72.85 years old (SD = 6.13, range = 60-84 years) for the short fusion group (P = 0.654). The long fusion group included 19 males (79.2%), and the short fusion group included 16 males (80.0%) (P = 1.000). The observation period was 25.58 (SD = 33.13) months for the long fusion group and 17.65 (SD = 26.12) months for the short fusion group (P = 0.390). There were no notable differences in SIN scores between the groups (P = 0.589). The results showed no significant differences in patient background or surgery-related factors between the two groups.

**Table 1:** Patient characteristics. We performed a Fisher's exact test to compare the two groups. * Sinonasal adenocarcinoma (4.2%) and urinary cancer (4.2%). SD, standard deviation; BMI, body mass index; ASA-PS, American Society of Anesthesiologists physical status; SIN, spinal instability neoplastic; ECOG PS, Eastern Cooperative Oncology Group performance status; KPS, Karnofsky Performance Scale.

Characteristic	Long fusion (n = 24)	Short fusion (n = 20)	P-value
Age, mean, years (SD)	73.75 (6.94)	72.85 (6.13)	0.654
Sex (male), n (%)	19 (79.2)	16 (80.0)	1.000
Observation period, months (SD)	25.58 (33.13)	17.65 (26.12)	0.390
BMI (kg/m^2^), mean (SD)	19.77 (3.21)	21.54 (4.07)	0.114
ASA-PS, n (%)			0.363
1	4 (16.7)	5 (25.0)	
2	17 (70.8)	10 (50.0)	
≥3	3 (12.5)	5 (25.0)	
Frankel grade, n (%)			0.753
B	3 (12.5)	1 (5.0)	
C	5 (20.8)	7 (35.0)	
D	5 (20.8)	3 (15.0)	
E	11 (45.8)	9 (45.0)	
Smoking, yes, n (%)			0.072
Past	12 (54.5)	3 (23.1)	
Current	4 (18.2)	1 (7.7)	
Alcohol habit, yes, n (%)			0.371
Past	8 (33.3)	3 (15.0)	
Current	7 (29.2)	4 (20.0)	
Chemotherapy			
Preoperative, n (%)	7 (29.2)	11 (55.0)	0.125
Postoperative, n (%)	12 (50.0)	15 (75.0)	0.124
Radiation therapy			
Preoperative, n (%)	4 (16.7)	4 (20.0)	1.000
Postoperative, n (%)	11 (45.8)	10 (50.0)	1.000
New Katagiri score (SD)	3.54 (1.79)	2.70 (1.45)	0.099
SIN score (SD)	10.38 (2.68)	9.95 (2.44)	0.589
Tokuhashi score (SD)	8.71 (2.79)	9.95 (2.68)	0.142
Charlson Comorbidity Index (SD)	8.88 (1.45)	9.10 (1.65)	0.633
ECOG PS, n (%)			0.406
0	2 (8.3)	0 (0.0)	
1	5 (20.8)	8 (40.0)	
2	6 (25.0)	5 (25.0)	
3	10 (41.7)	5 (25.0)	
4	1 (4.2)	2 (10.0)	
KPS, n (%)			0.878
30	1 (4.2)	1 (5.0)	
40	6 (25.0)	4 (20.0)	
50	5 (20.8)	4 (20.0)	
60	1 (4.2)	1 (5.0)	
70	1 (4.2)	1 (5.0)	
80	4 (16.7)	7 (35.0)	
90	4 (16.7)	2 (10.0)	
100	2 (8.3)	0 (0.0)	
Cancer type			0.466
Breast cancer	1 (4.2)	1 (5.0)	
Lung cancer	7 (31.8)	2 (10.0)	
Prostate cancer	2 (8.3)	4 (20.0)	
Lymphoma	1 (4.2)	2 (1.0)	
Thyroid cancer	1 (4.2)	2 (10.0)	
Kidney cancer	3 (12.5)	3 (15.0)	
Colorectal cancer	2 (8.3)	2 (10.0)	
Gastric cancer	2 (8.3)	0 (0.0)	
Pancreatic cancer	1 (4.2)	0 (0.0)	
Hepatocellular cancer	0 (0.0)	3 (15.0)	
Unknown primary	2 (8.3)	1 (5.0)	
Other*	2 (8.3)*	0 (0.0)	

Clinical outcomes

**Figure 2:**
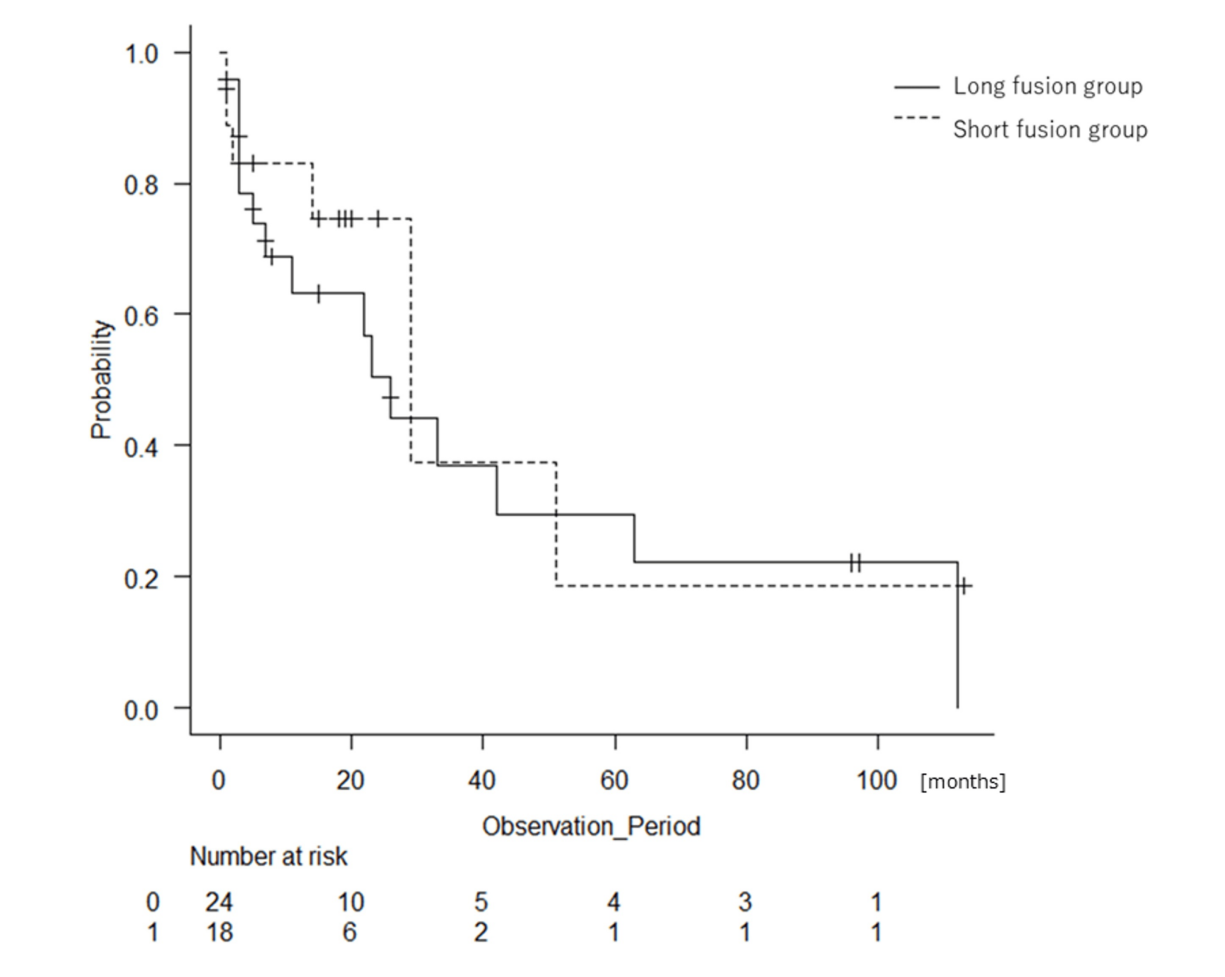
Kaplan-Meier curves showing the survival rates of the patients with fixation of four or fewer vertebrae (short fusion group: dotted line) or fixation of five or more vertebrae (long fusion group: black line). Median survival durations are 29 and 26 months, respectively (P = 0.533).

Figure 2 shows Kaplan-Meier curves representing the survival curves of both groups. The long fusion group tended to have slightly poorer short-term survival, but the difference in survival was not significant (median survival was 29 months for the short fusion group and 26 months for the long fusion group, P = 0.533). The purpose of the surgery was similar between the groups, with 50% of surgeries aimed at the appearance of paralysis in both groups, and 29.2% (long fusion group) and 40.0% (short fusion group) aimed at pain relief (P = 0.832).

Surgical result

MIS was performed on seven patients in the long fusion group (29.2%) and two patients in the short fusion group (10.0%). The average duration of surgery was 198.96 (SD 51.36) minutes for the long fusion group and 184.25 (SD 86.71) minutes for the short fusion group, with no statistically significant difference (P = 0.824). There were no significant differences in the type of surgery, whether planned or emergency (P = 0.771), or in the site of metastasis between the long and short fusion groups (P = 1.000). The rates of drainage tube placement and bone grafting were also similar in both groups (both P = 1.000) (Table 2). Regarding the localization of metastatic lesions treated with surgery, the long fusion group had 12 cases in the semi-rigid spine (Th3-Th10), three cases in the mobile spine (C3-C6 or L2-L4), and nine cases in other regions. On the other hand, the short fusion group had 10 cases, two cases, and eight cases, respectively. The short fusion group had a significantly higher incidence of surgical complications (45.0% vs. 12.5%, P = 0.021), with nine of 20 patients in the short fusion group experiencing complications compared to three of 24 in the long fusion group. Although the difference was not statistically significant (P = 0.125), the short fusion group experienced a higher rate of postoperative complications (55.0%) compared to the long fusion group (29.2%). The specific types of complications included cerebrospinal fluid leakage, epidural hematoma, instrument breakage, instrument displacement, instrument loosening, worsening of neurological symptoms, and reoperations (Table 3).

**Table 2:** Operative variables. We performed a Fisher's exact test to compare the two groups. SD, standard deviation; ICU, intensive care unit; HCU, high care unit; Th, thoracic; C, cervical; L, lumbar; Oc, occipital; S, sacral; MIS, minimally invasive surgery.

Variable	Long fusion (n = 24)	Short fusion (n = 20)	P-value
Surgery duration, minutes (SD)	198.96 (51.36)	184.25 (86.71)	0.824
Anesthesia duration, minutes (SD)	296.88 (56.91)	297.80 (106.10)	0.971
ICU/HCU admission, yes (%)	4 (16.7)	2 (10.0)	1.000
Purpose of surgery, n (%)			0.832
Paralysis	14 (50.0)	10 (50.0)	
Pain relief	7 (29.2)	8 (40.0)	
Preventive	3 (12.5)	2 (10.0)	
Type of surgery, n (%)			0.771
Planned surgery	12 (50.0)	9 (45.0)	
Emergency surgery	12 (50.0)	11 (55.0)	
Site of metastasis, n (%)			1.000
Semi-rigid (Th3-Th10)	12 (50.0)	10 (50.0)	
Mobile spine (C3-C6, L2-L4)	3 (12.5)	2 (10.0)	
Junctional spine (Oc-C2, C7-Th2, Th11-L1, L5-S1)	9 (37.5)	8 (40.0)	
Placement of drainage tube, yes, n (%)	22 (91.7)	18 (90.0)	1.000
Bone graft, yes, n (%)	3 (12.5)	3 (15.0)	1.000
MIS, n (%)	7 (29.2)	2 (10.0)	0.150

**Table 3:** Surgery-related complications and other complications during follow-up. A Fisher's exact test was performed to compare the two groups.

Variables	Long fusion (n = 24)	Short fusion (n = 20)	P-value
Complications related to surgery (during hospitalization)	3 (12.5)	9 (45.0)	0.021
Complications related to surgery (during follow-up)	7 (29.2)	11 (55.0)	0.125
Cerebrospinal fluid leaking	1	0	
Epidural hematoma	1	0	
Instrument breakage	0	1	
Instrument displacement	0	1	
Instrument loosening	3	1	
Neurological symptom worsening	4	9	
Reoperation	2	4	
Other complications	5 (20.8)	3 (15.0)	0.710

**Figure 3:**
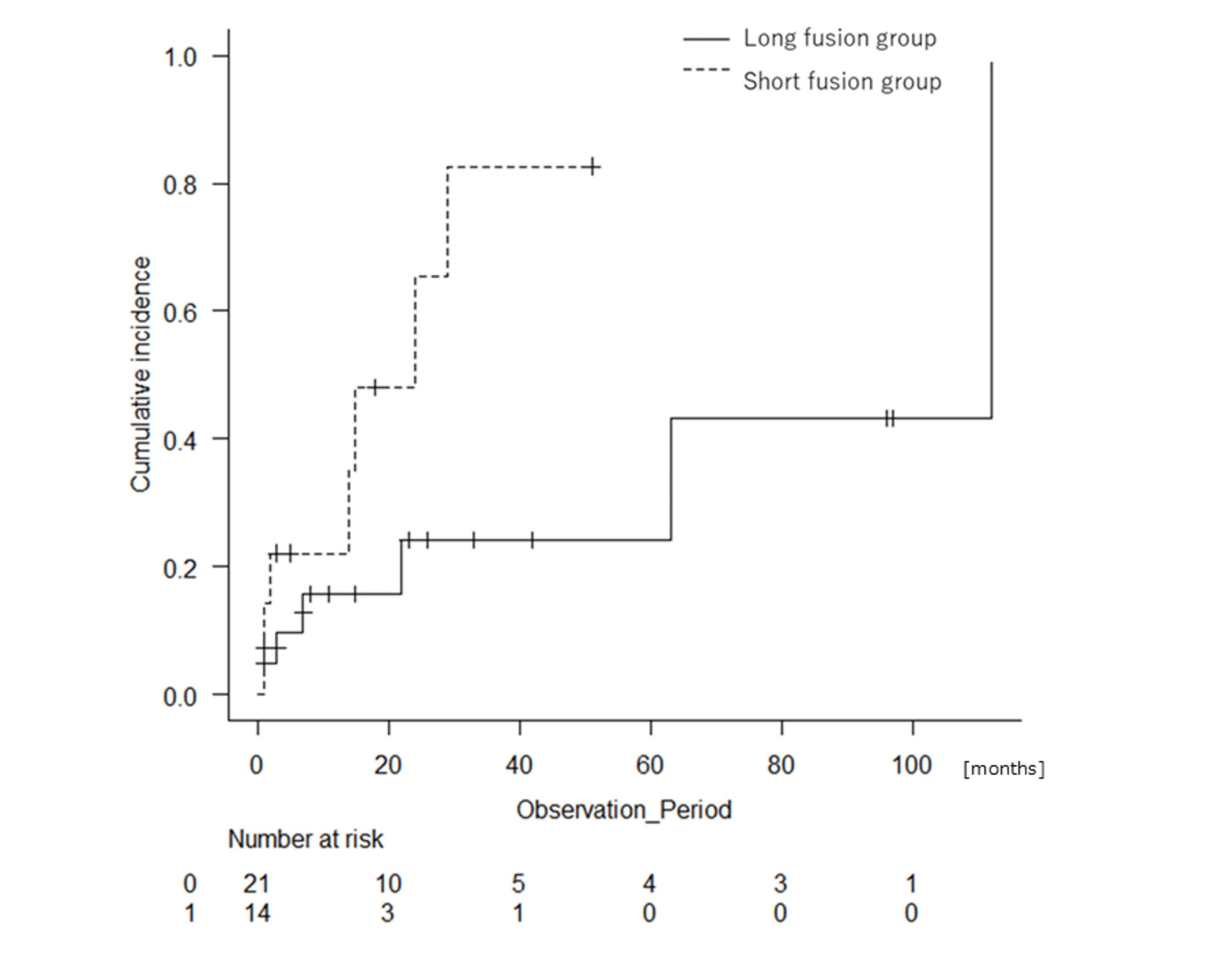
Cumulative incidence of the rates of complications related to surgery of the patients with fixation of four or fewer vertebrae (short fusion group: dotted line) or fixation of five or more vertebrae (long fusion group: black line) (P = 0.033).

**Figure 4:**
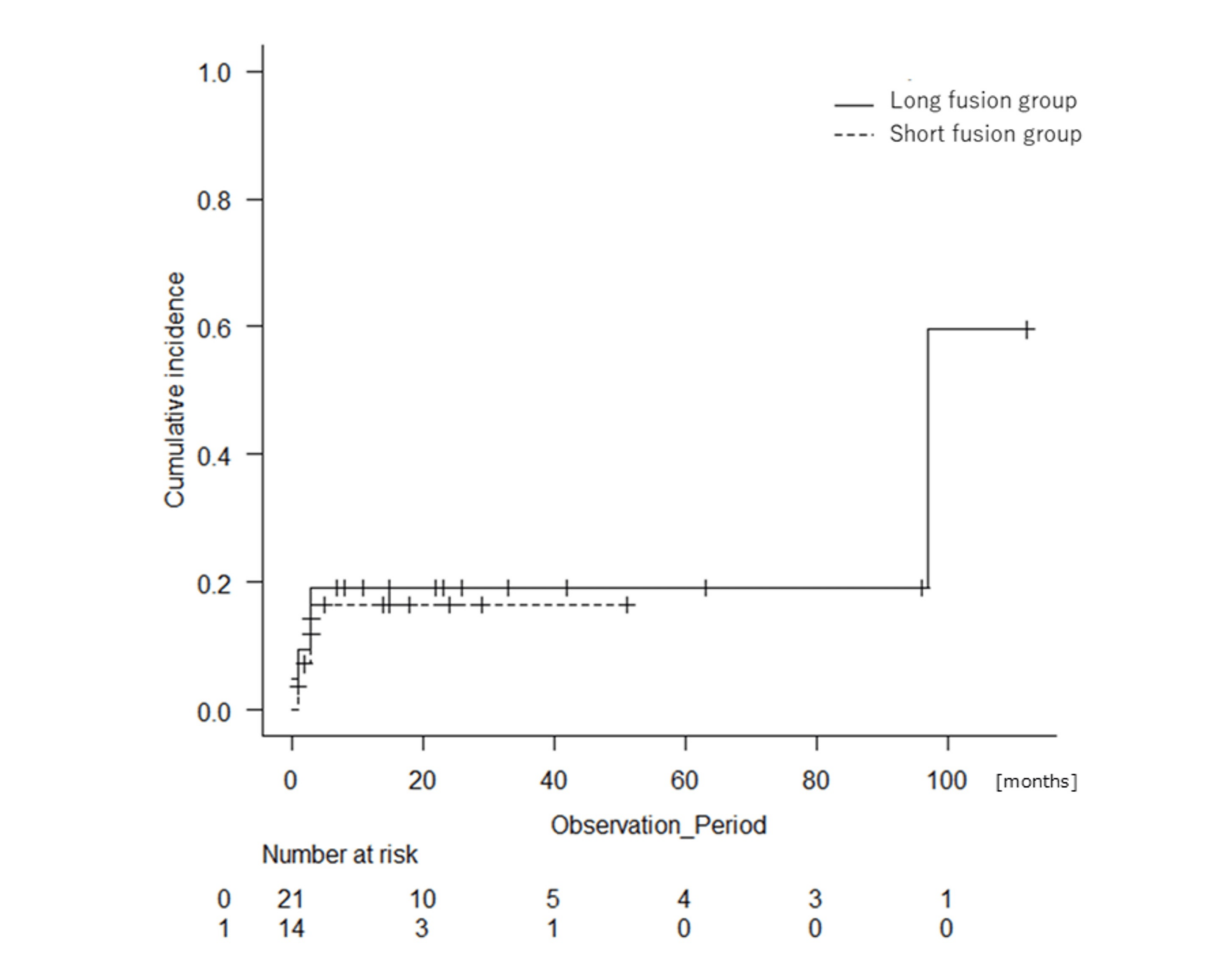
Cumulative incidence of the rates of other complications in the patients with fixation of four or fewer vertebrae (short fusion group: dotted line) or fixation of five or more vertebrae (long fusion group: black line) (P = 0.823).

Other complications during follow-up occurred in similar proportions between the two groups (20.8% for the long fusion group and 15.0% for the short fusion group, P = 0.710). They included delirium, nausea/vomiting, sepsis, swallowing disorder, and deep vein thrombosis. Complications include duplications in the same patient (Tables 4, 5 and Figures 3, 4).

**Table 4:** Number of complications in minimally invasive surgery. A Fisher's exact test was performed to compare the two groups.

Variables	Long fusion (n = 7)	Short fusion (n = 2)	P-value
Complications related to surgery	2	1	1.000
Other complication	1	0	1.000

**Table 5:** Number of complications in non-minimally invasive surgery. A Fisher's exact test was performed to compare the two groups.

Variables	Long fusion (n = 17)	Short fusion (n = 18)	P-value
Complications related to surgery	5	9	0.305
Other complication	4	3	0.691

## Discussion

In this study, surgical outcomes for spinal metastases in elderly patients were compared between a group requiring fusion of four or fewer vertebrae (short fusion group) and a group requiring fusion of five or more vertebrae (long fusion group) for lesions involving single-level vertebra. The results showed that the long fusion group had a significantly lower rate of postoperative complications than the short fusion group (12.5% vs. 45.0%). The most common complication observed in both groups was the worsening of neurological symptoms, followed by instrument loosening. MIS tended to be performed more frequently in the long fixation group than in the short fusion group (P = 0.150).

Our results suggested that longer fusion is effective in enhancing spinal stability in elderly patients. Particularly for elderly individuals, who are often affected by osteoporosis and have more fragile bones [20], longer fusion may prevent postoperative instability and could potentially reduce the worsening of neurological deficits. Although a past study showed that complications increase with an increase in the number of fixed vertebrae [11], there have been no similar reports regarding metastatic spinal tumors. The results of our study suggest that it may be important to obtain spinal stability by longer fusion to avoid neurological worsening.

Recently, MIS has been used with less invasiveness and better results [21,22]. We analyzed the occurrence of complications in MIS and non-MIS (Tables 4, 5). No differences were observed in postoperative complications between long fusion and short fusion in the MIS group (two in the long fusion group and one in the short fusion group). In the non-MIS group, there was a tendency toward a higher incidence of surgery-related complications in the short fusion group compared to the long fusion group (five in the long fusion group and nine in the short fusion group). These results indicate that in non-MIS, longer fixation may reduce postoperative complications. We considered MIS to be an effective method for reducing invasiveness based on the previous reports, although the number of cases was small in our study. Based on the results of this study, although this study has a small sample size, extending the fixation range using MIS may be an effective approach and it could be considered in surgical decision-making.

Other important considerations include the need for postoperative treatment of the primary tumor, which may continue after surgery. It will be necessary to discuss the treatment plan with physicians in other departments [20,23] and increase the levels of rest and activity as soon as possible after surgery. The present results showed complications of implant loosening and breakage, which may be due to the degree of postoperative rest the patient received in addition to the original bone fragility. For this reason, it is necessary for other professions to determine the level of bed rest and treatment according to the surgical procedure after surgery.

Limitations

We recognize the limitations of this study. First, our study was retrospective, and we divided the patients into two groups by range of fixation. The choice of technique was left to the surgeon, but the technique used can affect the outcome. Second, the number of cases included in this analysis was fewer than expected due to missing data and other factors. The inclusion of more cases may lead to the identification of independent prognostic factors relating to complications or survival. Furthermore, although the approximate lesion sites were classified, the specific vertebral levels were not precisely categorized, which could potentially affect the fixation range in certain cases. Third, this study did not measure bone density, which limited our ability to evaluate the relationship between bone density and fixation strength.

## Conclusions

This study compared surgical outcomes in elderly patients undergoing short fusion (four or fewer vertebrae) and long fusion (five or more vertebrae) for single-level spinal metastases. The results demonstrated no significant difference in survival rates between short and long fixation in elderly patients undergoing surgery for single-level spinal metastasis. In spinal metastasis surgery for elderly patients, extending the fixation range may be considered if necessary. Longer fusion was suggested to provide better spinal stability, particularly in elderly patients with fragile bones, potentially reducing the risk of postoperative neurological deterioration. While MIS showed promise in reducing invasiveness, its influence on complications was limited by the small sample size. The findings highlight the importance of achieving spinal stability through extended fixation to improve postoperative outcomes in elderly patients with spinal metastases. On the other hand, this study had a small sample size, and considering concerns such as tumor recurrence, a more careful selection of surgical procedures may be necessary for certain cases. Further research is needed in the future.
